# A metabolic biosignature of early response to anti-tuberculosis treatment

**DOI:** 10.1186/1471-2334-14-53

**Published:** 2014-01-31

**Authors:** Sebabrata Mahapatra, Ann M Hess, John L Johnson, Kathleen D Eisenach, Mary A DeGroote, Phineas Gitta, Moses L Joloba, Gilla Kaplan, Gerhard Walzl, W Henry Boom, John T Belisle

**Affiliations:** 1 Mycobacteria Research Laboratories, Department of Microbiology, Immunology and Pathology, Colorado State University, Fort Collins, CO 80523, USA; 2 Department of Medicine, Tuberculosis Research Unit (TBRU), Case Western Reserve University, Cleveland, OH 44106, USA; 3 Uganda-Case Western Reserve University Research Collaboration, Kampala, Uganda; 4 Department of Pathology, University of Arkansas for Medical Sciences, Little Rock, AR 72205, USA; 5 Department of Medical Microbiology, University School of Biomedical Sciences, College of Health Sciences, Makerere University, Kampala, Uganda; 6 Public Health Research Institute Center, University of Medicine and Dentistry of NJ, Newark, New Jersey 07103, USA; 7 DST/NRF Centre of Excellence for Biomedical Tuberculosis Research and MRC Centre for Molecular and Cellular Biology, Division of Molecular Biology and Human Genetics, Faculty of Medicine and Health Sciences, Stellenbosch University, PO Box 19063, Francie van Zijl Drive, Tygerberg 7505, South Africa

**Keywords:** Tuberculosis, Metabolomics, Biomarker, Mass spectrometry, Small molecule biosignature, Anti-tuberculosis therapy, *Mycobacterium tuberculosis*, Urine

## Abstract

**Background:**

The successful treatment of tuberculosis (TB) requires long-term multidrug chemotherapy. Clinical trials to evaluate new drugs and regimens for TB treatment are protracted due to the slow clearance of *Mycobacterium tuberculosis (Mtb)* infection and the lack of early biomarkers to predict treatment outcome*.* Advancements in the field of metabolomics make it possible to identify metabolic profiles that correlate with disease states or successful chemotherapy. However, proof-of-concept of this approach has not been provided for a TB-early treatment response biosignature (TB-ETRB).

**Methods:**

Urine samples collected at baseline and during treatment from 48 Ugandan and 39 South African HIV-seronegative adults with pulmonary TB were divided into discovery and qualification sets, normalized to creatinine concentration, and analyzed by liquid chromatography-mass spectrometry to identify small molecule molecular features (MFs) in individual patient samples. A biosignature that distinguished baseline and 1 month treatment samples was selected by pairwise t-test using data from two discovery sample sets. Hierarchical clustering and repeated measures analysis were applied to additional sample data to down select molecular features that behaved consistently between the two clinical sites and these were evaluated by logistic regression analysis.

**Results:**

Analysis of discovery samples identified 45 MFs that significantly changed in abundance at one month of treatment. Down selection using an extended set of discovery samples and qualification samples confirmed 23 MFs that consistently changed in abundance between baseline and 1, 2 and 6 months of therapy, with 12 MFs achieving statistical significance (p < 0.05). Six MFs classified the baseline and 1 month samples with an error rate of 11.8%.

**Conclusions:**

These results define a urine based TB-early treatment response biosignature (TB-ETRB) applicable to different parts of Africa, and provide proof-of-concept for further evaluation of this technology in monitoring clinical responses to TB therapy.

## Background

The emergence of multidrug resistant strains of *Mycobacterium tuberculosis (Mtb)* underscores the need for new drugs and shorter regimens to treat tuberculosis (TB), one of the world’s most prevalent infectious diseases. Considerable advancements have been realized in anti-TB drug development, but clinical evaluation of new drugs remains a protracted process [1,2]. Phase 2 clinical trials typically use patient conversion to negative-sputum culture after two months of treatment as a biomarker to assess therapeutic efficacy. However, this measure lacks the robustness needed to evaluate smaller numbers of patients in each arm of early clinical trials, or to evaluate treatment shortening regimens in Phase 3 trials [1,3]. Therefore, alternative, quantitative biomarkers that reliably measure a patient’s response to anti-TB treatment after a short period of time and serve as surrogate endpoints are needed to accelerate clinical trials [4,5].

Disease and inflammatory states correlate with changes in the biochemistry of a system (host and pathogen), and metabolomic approaches provide a direct measure of a systems biochemical profile [6-8]. Additionally, a diseased-state metabolic profile would be expected to revert to a normal-state (non-diseased) in response to successful treatment. Modern analytical platforms such as mass spectrometry (MS) provide accurate methods for assessing complex metabolic profiles, and when combined with multivariate statistical analyses, MS can elucidate time related metabolic changes that correlate with transition from a diseased- to a normal-state [7,9,10]. Metabolic flux has been exploited in the study of oncology, diabetes and cardiovascular disease, but has not been widely applied to infectious disease biomarker development [11-13]. We hypothesized that metabolomic analyses by liquid chromatography (LC)-MS of clinical samples collected at the time of TB diagnosis and at various treatment time points would reveal a metabolic flux that could be developed as a biosignature of treatment response.

Sputum and serum are conventional clinical specimens used for TB diagnosis and evaluation of TB treatment response [14,15]. However, urine is an alternative, non-invasive clinical sample, and has been successfully used to discover biomarkers for other diseases [11,16]. Urine contains a sizable fraction of the human metabolome and metabolites of microbial origin, and requires minimal processing for analysis by LC-MS [17-20]. Our efforts applied LC-MS to archived urine specimens collected as part of observational studies of TB patients undergoing standard therapy for pansusceptible, pulmonary TB. This resulted in the definition of a urine metabolite based TB-early treatment response biosignature (TB-ETRB) that measured a significant metabolic flux as early as one month of treatment.

## Methods

### Clinical samples

Anonymized archived urine samples used in the current studies were procured from the Tuberculosis Research Unit (TBRU) and Stellenbosch University. The samples provided by the TBRU originated from the NAA2m study (DMID 08–0023) conducted in Uganda (http://www.case.edu/affil/tbru/research_naa2m.html). This was a prospective observational cohort study of adults with newly-diagnosed sputum smear-positive, culture-confirmed pulmonary TB treated with supervised standard chemotherapy. Samples were collected from 48 patients at the time of TB diagnosis (D0) and month-1 (M1), month-2 (M2) and month-6 (M6) of treatment. The Stellenbosch University samples were from 39 patients from the Action TB Surrogate Marker study [21] and the D0, M1 and M6 time point samples were evaluated. All urine specimens were from adult pulmonary cavitary TB patients of both sexes without HIV co-infection. Urine specimens were stored at -80°C upon collection. All patients successfully responded to anti-TB therapy.

Study NAA2m (DMID 08–0023; A Pilot Study to Evaluate Nucleic Acid Amplification (NAA) and Other Tests to Predict Relapse of Tuberculosis and to Monitor the Effectiveness of Treatment) was conducted according to the Helsinki Declaration and International Conference on Harmonisation guidelines. There was no remuneration or financial incentive for participation. Individual written informed consent was obtained for study participation, HIV testing, and permission for blood and other samples to be stored and used in future TB studies. The study was approved by the institutional review boards of the U.S. Centers for Disease Control (IRB B), the University Hospitals Case Medical Center IRB (FWA00003937) and the Joint Clinical Research Centre Review Committee (IRB00002647), which is duly constituted under the supervision of the Uganda National Council for Science and Technology (FWA00001293; http://www.uncst.go.ug/) and the Office of the President of Uganda.

The Action TB study was conducted according to the Helsinki Declaration and International Conference on Harmonisation guidelines. There was no remuneration or financial incentive for participation. Individual written informed consent was obtained for study participation and HIV testing. The study “A prospective evaluation of surrogate markers for disease and relapse or reinfection in adult patients with pulmonary tuberculosis” was approved by the Stellenbosch University Health Research Ethics Committee (Ref no 99/039) and Cape Town City Health.

### LC-MS analysis of urine samples

**Figure 1:**
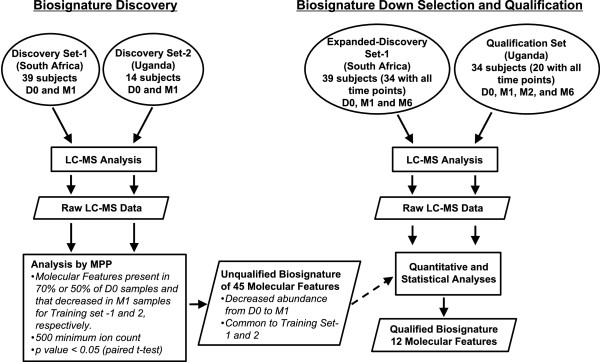
**Work flow for the development and qualification of a urine metabolite biosignature of TB treatment response.** Biosignature discovery (left) utilized D0 and M1 samples from two distinct geographical locations. This yielded 45 MFs that decreased in abundance between D0 and M1. Biosignature down selection and qualification (right) also used samples from two geographically distinct locations and expanded the analyses to additional time points after the start of anti-TB therapy.

The D0, M1 and M6 samples from Stellenbosch University and the D0 and M1 samples from 14 patients of NAA2m study were used as discovery sets. Samples from all time points of the remaining 34 individuals of the NAA2m study were used as a qualification set. Samples used in the discovery phase (Figure 1) were sterilized by γ-irradiation. Those used in the down selection and qualification phase (Figure 1) were not γ-irradiated and additional biosafety measures were taken in handling these. The creatinine concentration of each sample was measured by an alkaline picrate based colorimetric assay (Oxford Biomedical Research, Oxford, MI). LC-MS analyses of urine samples were performed following the methods described in Mahapatra et al. [22]. Positive-ion MS data in both centroid and profile modes were collected using the Agilent MassHunter Data Acquisition software. Data for the discovery samples sets were obtained in the 4 GHz high-resolution modes and that used in down selection and qualification was obtained in the 2 GHz extended dynamic range mode.

### Data analyses

LC-MS data from the discovery and qualification sample sets were processed with the Molecular Feature Extractor (MFE) algorithm tool of the Agilent MassHunter Qualitative Analysis software version B.04.00. The parameters used for MFE were minimum 500 counts, ion species H^+^, charge state maximum 1, compound ion count threshold 2 or more ions, and all other parameters were default settings. Comparative analyses of the MFE data files from the training sample sets were performed with the Agilent Mass Profiler Pro (MPP) software version B. 02.01. The molecular features (MFs) were aligned with a 0.4 min retention time and 15 ppm mass tolerance. Aligned MFs were filtered based on their presence in 50%, 60%, or 70% of samples in at least one time point group. The relative abundance of MFs obtained from different treatment time points were compared and features that varied significantly by at least 2-fold based on pairwise t-test or ANOVA (p <0.05) were selected. The MFs that decreased significantly in abundance between D0 and M1 for discovery set-1 and -2 (Figure 1) were selected by *k*-means clustering analysis. The abundances of selected MFs (area under the peak for the monoisotopic mass) in the extended discovery and qualification data sets were determined using the Agilent MassHunter quantitative analysis software and exported as a Microsoft Excel spreadsheet for statistical analyses.

### Statistical analyses

Statistical analysis was performed using SAS version 9.2. All feature intensity (arbitrary numbers) values were transformed to the log2 scale prior to statistical analysis. To prevent undefined values, a value of one was added to all values prior to transformation. Separately for each MF of the Stellenbosch University and NAA2m data sets, a repeated measures analysis was performed using SAS proc mixed. The response variable was log2 transformed intensity (abundance) value from the Agilent MassHunter quantitative analysis software. The model included a fixed effect for time and a random subject effect to account for repeated measures. Comparisons across time points were based on contrasts of the model. After identifying a subset of features with consistent changes across time and sample sets, logistic regression was used to model treatment status (D0 versus M1).

### Identification of the metabolites

The chemical formulas of the MFs were predicted from the accurate mass data using the molecular formula generator tool of Agilent MassHunter Qualitative Analysis software. These molecular formulas were searched against the Metlin compound database (Metlin_AMRT_PCDL.cdb) using the ID browser application of MPP. The same molecular formulas were also searched against publicly available Human Metabolome Database (HMDB, http://hmdb.ca) [23].

## Results

### Clinical sample evaluation

To allow development and assessment of a robust biosignature, urine samples from adult pulmonary cavitary TB patients of both sexes without HIV co-infection, collected at the initial time of TB diagnosis (D0) (i.e. before start of therapy), and at different treatment time points during the course of therapy were divided into discovery and qualification sets (Figure 1). To minimize the impact of confounding variables (i.e. normal flora, diet, racial ethnicity, age, and sex), samples from two geographical regions (Uganda, 48 patients; and South Africa, 39 patients) were analyzed and the data normalized to urinary creatinine levels [24].

**Figure 2:**
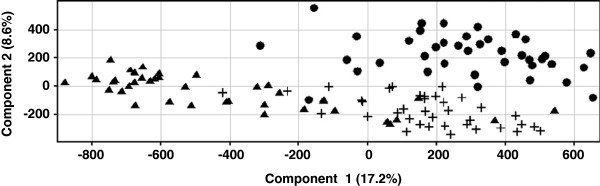
**Unsupervised PCA of D0 (triangle), M1 (circle), and M6 (cross) samples from South African patients.** The PCA was constructed based on MFs that were present in at least 60% of the samples for any given time point and differed in abundance between time points by at least 2 fold with a p < 0.05.

We initially applied LC-MS analyses and data processing as described in Materials and Methods to an expanded-discovery set of urine samples from South Africa (D0, M1 and M6) to assess the separability of these time-grouped samples based on metabolic signatures. Specifically, an ANOVA was performed to identify relevant MFs (ions with defined accurate mass and retention time) present in at least 60% of the samples for any given treatment time point and that differed in abundance between time points by at least 2 fold (log2 data). An unsupervised principal component analysis (PCA) demonstrated that D0 samples separated from those of the M1 and M6 treatment time points (Figure 2). However, the data also revealed a separation between the M1 and M6 samples. This indicated that metabolic profiling can be utilized to assess anti-TB treatment, but that the selection of a TB-ETRB based on samples from three time points would be difficult to deconvolute.

### Biosignature discovery

**Figure 3:**
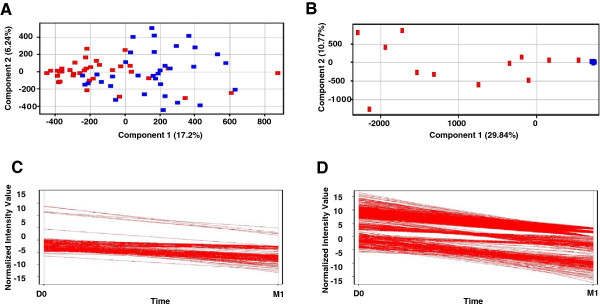
**Unsupervised PCA of D0 (triangle) and M1 (circle) patient samples, and MFs demonstrating a decrease in abundance after the start of anti-TB treatment.** The PCAs were constructed based on MFs present in at least 70% and 50% of the samples for any given time point of Discovery Set-1 **(A)** and Discovery Set-2 **(B)**, respectively, and that decreased in abundance between D0 and M1 by at least 2 fold with a p < 0.05. A *k*-means clustering analysis was performed to select MFs that decreased in abundance between D0 and M1 of Discovery Set-1 **(C)** and Discovery Set-2 **(D)**.

To allow selection of a TB-ETRB, pairwise analyses of LC-MS data of D0 and M1 samples from Discovery Sets-1 and -2 were performed (Figure 1). This analysis was performed with the Agilent MPP software that provides a method to filter through large data sets for MFs that differ between groups (i.e. D0 and M1) based on the MF abundance as measured by peak height and normalized to the mean of all samples, as well as user provided parameters. This resulted in the identification of 822 and 2644 MFs, respectively, for Discovery Sets-1 and -2 that either decreased or increased in abundance (2 fold) following the onset of treatment. PCA based on the selected MFs demonstrated discrete clustering of the D0 and M1 samples of both discovery sets (Additional file 1). As a means of validating the overall approach, the predicted metabolites of anti-TB drugs [25-29] were identified based on accurate mass measurements. These compounds were included in the group of MFs that increased in abundance between the D0 and M1 samples (data not shown). To avoid biasing a TB-ETRB by inclusion of known or unknown drug metabolites, subsequent analyses were limited to MFs that decreased in abundance following the onset of treatment (Figure 3). These data demonstrated that the separation of D0 and M1 samples was more pronounced for Discovery Set-2 versus Discovery Set-1. Further, the inclusion of MFs that changed abundance in either direction (Additional file 1) versus those that only decreased in abundance (Figure 3) did not alter this trend between discovery sets. From the 183 and 659 MFs that decreased in abundance between D0 and M1 for Discovery Sets-1 and -2, respectively, 26 differentiating MFs were consistent between the two data sets. An additional 19 MFs were included for their high significance in a single discovery set to give the initial discovery-phase TB-ETRB of 45 MFs (Figure 1).

### Biosignature down selection and qualification

Application of a MS defined metabolic biosignature to assess treatment response during clinical trials would be facilitated by the evaluation of LC-MS data in a manner that was independent of a specific software platform. With this in mind further downs selection and qualification of the 45-MF TB-ETRB was performed on quantitative data (area under the peak for the selected MFs) directly extracted from LC-MS files of urine specimens in Expanded Discovery Set-1 and the Qualification Set (Figure 1). It is noted that the Expanded Discovery Set-1 includes the D0 and M1 samples used in the initial TB-ETRB discovery phase, whereas the Qualification Set samples were analyzed for the first time during the down selection and qualification phase. The inclusion of the Expanded Discovery Set-1 was justified in these later evaluation steps since the more robust quantitative measure of MF peak area was used with the Agilent Quantitative Analysis software. This was in contrast to the discovery phase that was based on the high-throughput filtering power of the MPP software with MF abundance based on peak height.

The quantitative data of each MF were evaluated for change in abundance across the D0, M1, and M6 time points for Expanded Discovery Set-1; and D0, M1, M2, and M6 samples for the Qualification Set. Ten of the 45 MFs failed to yield extractable quantitative data from the LC-MS files of the Qualification Set. Thus, the biosignature that could be further down selected based on robustness across multiple data sets was comprised of 35 MFs. The molecular formulas of these 35 MFs were predicted based on their accurate masses and interrogated against the Human Metabolome and METLIN databases to provide putative structure identification of the MFs (Additional file 2).

**Figure 4:**
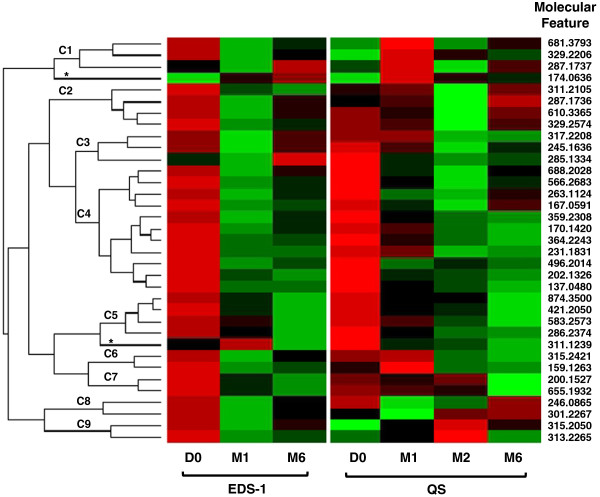
**Heat map of 35 MFs showing the change in abundance across multiple time point.** A cluster analysis was performed on log2 transformed averaged abundances of individual MFs at each time point for samples of Expanded Discovery Set-1 (EDS-1) and the Qualification Set (QS). MFs are displayed in rows and noted by the monoisotopic mass of the MF. Columns display time points. Visual analysis of the heat map revealed four clusters (C1, C3, C6, C9) of MFs that behaved dissimilarly between qualification sets; five clusters (C2, C4, C5, C7, C8) of MFs that behaved similarly between qualification sets; and two MFs (*) that did not fit into a specific cluster because of the low abundance at D0. Red indicates high abundance, green indicates low abundance.

Hierarchical clustering based on the abundance of the 35 individual MFs across all time points of the Expanded Discovery Set-1 and Qualification Set was performed. This demonstrated nine clusters of MFs based on their kinetics of change in abundance, and two MF (174.0636, and 311.1239) with abundance changes that excluded them from any of the clusters (Figure 4). The MFs forming clusters C1, C3, C6 and C9 behaved inconsistently between the two sample sets, with the most relevant inconsistency occurring in abundance change from D0 to M1. A total of 23 MFs that typically displayed consistent abundance change trends across treatment time points of both the Expanded Discovery Set-1 and Qualification Set were encompassed by clusters C2, C4, C5, C7 and C8. As expected the MFs in these five clusters decreased in abundance between the D0 and M1 time points, and either continued to show decreased abundance at later time points or slightly increased in abundance after the initial D0 to M1 decrease.

**Figure 5:**
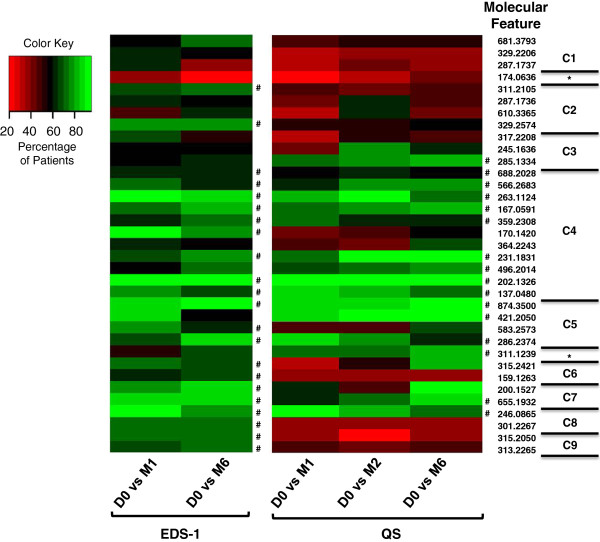
**Heat map based on the percentage of patients that demonstrated a decreased abundance in each of the 35 MFs between D0 and other time points.** The MFs are ordered based on the clusters identified in Figure 3, and the MF clusters are noted to the right of the individual MFs that are designated by their monoisotopic mass. The Expanded Discovery Set-1 (EDS-1) and the Qualification Set (QS) were evaluated separately for the percent of patients demonstrating a decrease in MF abundance. ^#^ indicates MFs that consistently decreased in abundance from D0 in at least 60% of the patients for all time points of a sample group. The exact patient percentages used to derive this graphical representation are provided in Additional file 3.

The above analyses were performed with values averaged across patient samples of each data set. To further assess robustness, each MF was evaluated on a patient-by-patient basis by calculating the percentage of patients demonstrating a decrease in abundance between the D0 and later time points. The Expanded Discovery Set-1 demonstrated that 91% to 35% of the patients yielded a decreased abundance of individual MFs between D0 and M1 (Figure 5 and Additional file 3). A similar range of these same patients (94% to 24%) revealed a decrease in the abundance of individual MFs between D0 and M6. Likewise, the Qualification Set displayed a decrease in MF abundance between D0 and M1, or D0 and M6 that ranged between 95% and 20%, or 95% and 40% of the patients, respectively (Figure 5 and Additional file 3). A total of 17 MFs decreased in abundance between D0 and other time points in 70% or greater of the patients in at least one of the sample sets, and five of these 17 MFs decreased in abundance in at least 70% of the patients from both sample sets. Three additional MFs from this group of 17 decreased in abundance between D0 and other time points in at least 60% of patients of both sample sets (Figure 5 and Additional file 3). All but three of the 17 MFs with the most consistent patient-to-patient abundance change were encompassed in cluster C2, C4, C5, C7 or C8 (Figure 4), further indicating that MFs in these clusters likely provide the most robust biosignature of treatment response. It was interesting to note that a large percentage of patients (at least 60%) from the Expanded Discovery Set-1 presented an increased abundance between D0 and other time points for MF 174.0636 and the majority of patients from the Qualification Set also showed this MF increasing in abundance over time. Thus, this outlier is truly a MF that tended to increase with treatment although the MPP software initially indicated it decreased in abundance. The MFs of C1, C3, C6 or C9 typically did not yield robust patient-to-patient results or produced dichotomous results between the two sample groups. This is consistent with the hierarchical cluster data (Figure 4) and the conclusion that these MFs would lack value in assessing treatment response.

**Figure 6:**
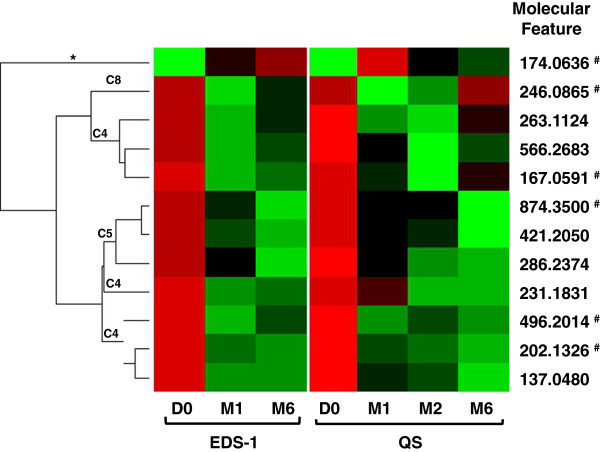
**Heat map of 12 MFs determined to have the highest statistical significance in both the Expanded Discovery Set-1 (EDS-1) and Qualification Set (QS).** The heat map displays change in abundance across multiple time point (columns) for each MF (rows). The MFs were arranged based on clustering analysis (left) and the associated clusters identified in Figure 4 are noted (C4, C5, C8 and *). ^#^indicates the molecular features identified by logistic regression analysis that classify D0 and M1 samples with an error rate of 11.8%.

To define a distinguishing TB-ETRB that could be applied at later anti-TB treatment time-points as well as M1, a repeated measures analysis was performed on the extracted quantitative data of the 35 MFs at all time points of the Expanded Discovery Set-1 and Qualification Set. This revealed 18 MFs with consistent and significant abundance changes between D0 and M1, and D0 and M6 for the Expanded Discovery Set-1 (Additional file 4). Of these 18 statistically significant MFs, 12 also showed consistent and significant changes in abundance between D0 and M1, and D0 and M2 or M6 of the Qualification Set (Table 1 and Figure 6). As expected, the repeated measures analyses showed that all 12 of the MFs except 174.0636 decreased in abundance between D0 and later time points. To further assess this 12 MF TB-ETRB, the D0 and M1 data of all 73 subjects used in the down selection and qualification phase were applied to a logistic regression model fitted to predict time status (D0 versus M1). Prior to model fitting, MF abundance values were log transformed and standardized to have mean zero and standard deviation of one for each MF and data set separately. Best subsets selection was used to identify the logistic regression model with the smallest AIC value, and resulted in the selection of six MFs (167.0591, 174.0636, 202.1326, 246.0865, 496.2014, and 874.3500) (Figure 6). Evaluation of the model based on leave-one-out cross-validation revealed an error rate of 11.8%. It is noted that since all of the TB patients evaluated in this study responded to treatment, this model has not been tested for prediction of successful treatment response. Nevertheless, this analysis provides proof-of-concept that a metabolite-based biosignature can be established to monitor anti-TB treatment as early as one month.

**Table 1:** Statistically significant MFs after down selection and qualification

**MF**^**1**^	**Predicted formula**^**2**^	**Alternate formula ±10 ppm**^**3**^	**DB Match**^**4**^
137.0484	C_7_H_7_NO_2_	None	p-Aminobenzoic acid + 10
167.0591	C_8_H_9_NO_3_	None	Pyridoxal/isopyridoxal + 10
174.0636	C_6_H_10_N_2_O_4_	C_8_H_15_PS	Formimino-L-glutamic acid + 1
202.1326	C_9_H_18_N_2_O_3_	None	Leu Ala + 8
231.1831	C_12_ H_25_ N O_3_	None	None
246.0865	C_9_H_14_N_2_O_6_	Multiple	L-alpha-Aspartyl-L-hydroxyproline + 2
263.1124	C_9_H_17_N_3_O_6_	Multiple	Thr Gly Ser + 8
286.2374	C_14_H_30_N_4_O_2_	None	N1,N12-Diacetylspermine + 1
421.2051	C_19_H_27_N_5_O_6_	Multiple	None
496.2014	C_25_H_28_N_4_O_7_	Multiple	None
566.2683	C_29_ H_48_ N_2_ O P_2_ S_2_	Multiple	None
874.3547	C_36_ H_65_ N_2_ O_16_ P_3_	Multiple	None

^1^The MF is denoted by its monoisotopic mass.

^2^The chemical formula was predicted based on accurate mass by molecular formula generator algorithm of Agilent MassHunter software.

^3^The alternate chemical formulas are within 10 ppm of the observed monoisotopic mass.

^4^The structure is the product with the closest match to the observed monoisotopic mass. The number denotes the number of structures in METLIN or HMDB databases that share the same molecular formula.

## Discussion

The present studies elucidated a small molecule metabolic biosignature that differentiates TB patients prior to the start of therapy from those successfully responding to treatment early (one month) after the start of therapy. Additionally, we demonstrated that a TB-ETRB can be developed using a non-invasive clinical specimen such as urine. Efforts to define diagnostic or prognostic biomarkers of TB have largely focused on microbiological and immunological measurements or identification of bacterial macromolecules [30-35]. Nevertheless, small molecule metabolites or metabolic processes such as adenosine deaminase activity in pleural fluid or hydroxyproline in urine are proposed as diagnostic markers of TB [36,37]. Moreover, several reports have evaluated the use of volatile organics in breath condensates as metabolic markers of TB [38-40]. Weiner et al. recently performed metabolomic analyses on sera from active and latent TB patients, as well as healthy controls to identify 20 small molecules as a diagnostic signature of TB [41]. Thus, as with non-infectious human diseases, [11-13] a growing body of evidence supports the use of small molecule metabolites as diagnostic or prognostic markers of TB.

Advanced computational tools greatly enhance the field of metabolomics and allow for the development of metabolic biosignatures [7]. Our metabolomic based analyses of urine allowed the separation of TB patients' samples based on treatment time points. However, it was noted that the profile of D0 versus M1 distinguishing metabolites likely differed from those allowing recognition of D0 versus M6 samples. Thus, a pairwise comparison of D0 and M1 samples and the subsequent filtering of the differentiating MFs such that they could be applied to other time points during therapy were adopted for the biosignature discovery phase. The first filtering step ensured patients from two distinct geographical regions shared the D0 versus M1 MFs. A second filtering step was used to remove potential bias introduced by drug metabolites and thus, the biosignature was limited to MFs that decreased in abundance after the start of therapy. This resulted in intermediate list of 45 MFs. The exclusion of MF that increased in abundance between D0 and M1 was a conservative approach for this proof-of-concept study. However, as we recently demonstrated with the discovery of an INH-NAD adduct in urine of TB patients undergoing treatment (22), there was a possibility that unknown drug metabolites might have biased biosignature data. Although this conservative approach can be justified for a proof-of-concept study, it must be recognized that MFs increasing in abundance with successful anti-TB treatment are likely to be equally valuable in predicting treatment response as long as they are shown not to be direct products of drug metabolism.

The 45 MFs from the discovery phase were down selected with LC-MS data from an expanded set of discovery samples and previously untested patient samples representing a qualification set. Moreover, the down selection was performed on the quantitative data extracted from the raw LC-MS files and was extended to treatment time points beyond M1. This process yielded 23 MFs that correlated well between the sample sets, and over half of these 23 MFs were determined to be statistically significant (p < 0.05) by a univariant analysis. Multivariant statistical modeling further selected six MFs that yielded an 11.8% error rate in classifying D0 and M1 samples. Although down selection based on consistency of data across sample sets and statistical analyses define the most robust MFs to include in a TB-ETRB, it is also important to understand why some of the 45 MFs from the discovery phase behaved inconsistently in evaluation of data for down selection and qualification. Firstly, not all patients will be at the same state of disease clearance at a specific treatment time point, thus the kinetics of abundance change for individual metabolites could vary from patient-to-patient during the course of treatment. Another possibility is the difference in handling of samples between the discovery and down selection phase . To assess samples in a manner that more closely resembles what might occur during a clinical trial, the Qualification Set samples were not subjected to γ-irradiation; a process that was applied to the other samples and resulted in elevated temperatures and potential alteration of some metabolites. Additionally, significant time gaps existed between the analyses of each sample set, and some MFs may have lacked a consistency that would withstand minor technical variability between analyses. While such variability in sample handling could exclude potentially useful MFs, it also allowed for selection of the most robust MFs. A third variable was the data analysis used for discovery versus that used in down selection. This likely explains why one MF (174.0636) selected during the discovery phase for decreased abundance between D0 and M1 was subsequently shown to increase in abundance after the start of treatment when LC-MS data were analyzed for down selection and qualification. The MPP software analyzes MFs that are selected based on ^12^C monoisotopic mass to ^13^C isotopic mass ratios; a process vulnerable to technical issues such as detector saturation [9,42]. Analyses performed with the LC-MS data for down selection, however, only considered the abundance (peak area) of the monoisotopic mass of each MF and data that was collected in an extended dynamic range mode; thus, eliminating detector saturation artifacts. This observation underscores the need and utility of identifying and correcting misclassifications via qualification studies and the use of unmodified quantitative MS data.

Bacteriological analysis of sputa is the most widely accepted method for the diagnosis and assessing treatment response of pulmonary TB [1]. In fact sputum culture results at two months of treatment are applied in Phase II clinical trials as an early indicator of drug efficacy. Analysis of individual molecules in sputa, however, can be technically challenging owing to viscosity and complexity of the matrix, as well as the absence of markers against which analyte abundance can be normalized. In contrast urine allows for facile recovery of analytes and can be normalized based on creatinine concentrations [24]. Metabolic profiles of individual urine samples will vary due to differences in diet, normal flora and other lifestyle related factors. Racial origin, age and sex will also influence metabolite profiles in sample subpopulations [43]. The use of samples from two geographical regions and the implementation of stringent biosignature selection criteria allowed for the identification of multiple MFs in the urine that changed consistently with early treatment response in both geographical regions. The data and approach presented provide proof-of-principle that a small-molecule, urine based TB-ETRB could be applied as a tool in clinical trials of new TB drugs and regimens.

This study also demonstrated that a metabolic flux occurs early during the successful treatment of TB patients. Thus, the metabolites identified based on abundance change over the course of treatment are likely indicators of diseased individuals returning to a normal or healthy state. This is underscored by the observation that the TB-ETRB behaved similarly at M1 and later time points of treatment, including M6. Additional studies, however, are needed to validate these biomarkers in patients with TB who are treated and followed for longer term unfavorable clinical outcomes such as treatment failure, recurrent TB or death, and for which microbiological data can be correlated with metabolic flux. Future analyses should also include patients from other geographical regions and patients co-infected with HIV as well as variations in infecting *Mtb* strains [44,45]. Likewise, fully elucidating the structural identity and biological significance of each MF can enhance the utility of a biosignature. Further studies are required to confirm the putative chemical identities assigned to the MFs of the TB-ETRB. This can be a protracted process [22,46,47], and one that is currently being pursued.

## Conclusions

Tuberculosis (TB) continues to have a major impact on global health, and the continued emergence of multiple drug resistant TB has enhanced the need for new anti-TB drugs. The need to accelerate anti-tuberculosis drug development and the study of new compounds in clinical trials is hindered by a lack of biomarkers that serve as indictors of clinical outcome or response to treatment. The current measure of treatment response is dependent on sputum culture methodology that can take weeks to obtain results and for which a clinical specimen becomes more difficult to collect as patients respond favorably to treatment. LC-MS analyses of aliquots of human urine normalized to creatinine concentrations allowed for the elucidation of a qualified small molecule (< 1,000 Da) metabolite biosignature comprised of 23 MFs that consistently changed in abundance over the course of treatment in these individuals; with 12 of these MFs achieving statistical significance. These studies and data demonstrated for the first time the feasibility of using small molecule biosignatures to monitor the response of individuals to anti-TB therapy. The analysis of clinical samples by LC-MS is rapid in comparison to the currently used culture methods for monitoring of anti-TB therapy and the qualified biosignature developed had a level of robustness that allowed for it to be applied in separate geographical settings. Moreover, this methodology was applied to urine; a clinical sample that requires little processing and is collected in a non-invasive manner.

## Abbreviations

HMDB: Human metabolome database; LC-MS: Liquid chromatography-mass spectrometry; MPP: Mass profiler pro; MS: Mass spectrometry, MFs, Molecular features; MFE: Molecular feature extractor; M1: Month-1; M2: Month-2; M6: Month-6; Mtb: *Mycobacterium tuberculosis*; PCA: Principal component analysis; D0: Time of TB diagnosis; TB: Tuberculosis; TB-ETRB: TB-early treatment response biosignature; TBRU: Tuberculosis Research unit.

## Competing interests

AMH, GK, GW, KDE, JLJ, MAD, MLJ, PG, and WHB have no competing interests. JTB and SM have filed an invention disclosure with Colorado State University covering the biomarkers described in this manuscript and Colorado State University is the employer of JTB and SM and will decide whether or not to file a patent on the biomarkers described.

## Authors’ contribution

SM performed the mass spectrometry; SM, AMH, MAD and JTB performed statistical and or data analyses; JlJ, GW, KDE, PG, MLJ and WHB provided coordination, collection and distribution of clinical samples and the associated data; SM, JTB, WHB, GK, KDE, JLJ, and GW all participated in and significantly contributed to the overall study design; JTB and SM were responsible for directing the science and writing of the manuscript; MAD, AH, GK, WHB, JLJ, and GW contributed significantly in the editing of the final manuscript. All authors read and approved the final manuscript.

## Pre-publication history

The pre-publication history for this paper can be accessed here:

http://www.biomedcentral.com/1471-2334/14/53/prepub

## Supplementary Material

Additional file 1 **Unsupervised PCA of D0 (triangle) and M1 (circle) samples.** The PCAs were constructed based on MFs present in at least 70% and 50% of the samples for any given time point of Discovery Set-1 (A) and Discovery Set-2 (B), respectively, and that differed in abundance between time points by at least 2 fold with a p<0.05. Click here for file

Additional file 2 MFs of Biosignature Used for Qualification. Click here for file

Additional file 3 Per Patient Evaluation of MF Robustness. Click here for file

Additional file 4 Results of Repeated Measures Analysis. Click here for file
