# Supplementary material for: A metabolic biosignature of early response to anti-tuberculosis treatment
Source: BMC Infect Dis. 2014 Jan 31;14:53. doi: 10.1186/1471-2334-14-53 (PMC3918231; doi:10.1186/1471-2334-14-53)
Supplement: Additional file 1 — Unsupervised PCA of D0 (triangle) and M1 (circle) samples. The PCAs were constructed based on MFs present in at least 70% and 50% of the samples for any given time point of Discovery Set-1 (A) and Discovery Set-2 (B), respectively, and that differed in abundance between time points by at least 2 fold with a p<0.05. [file 1471-2334-14-53-S1.pptx]

## Slide 1
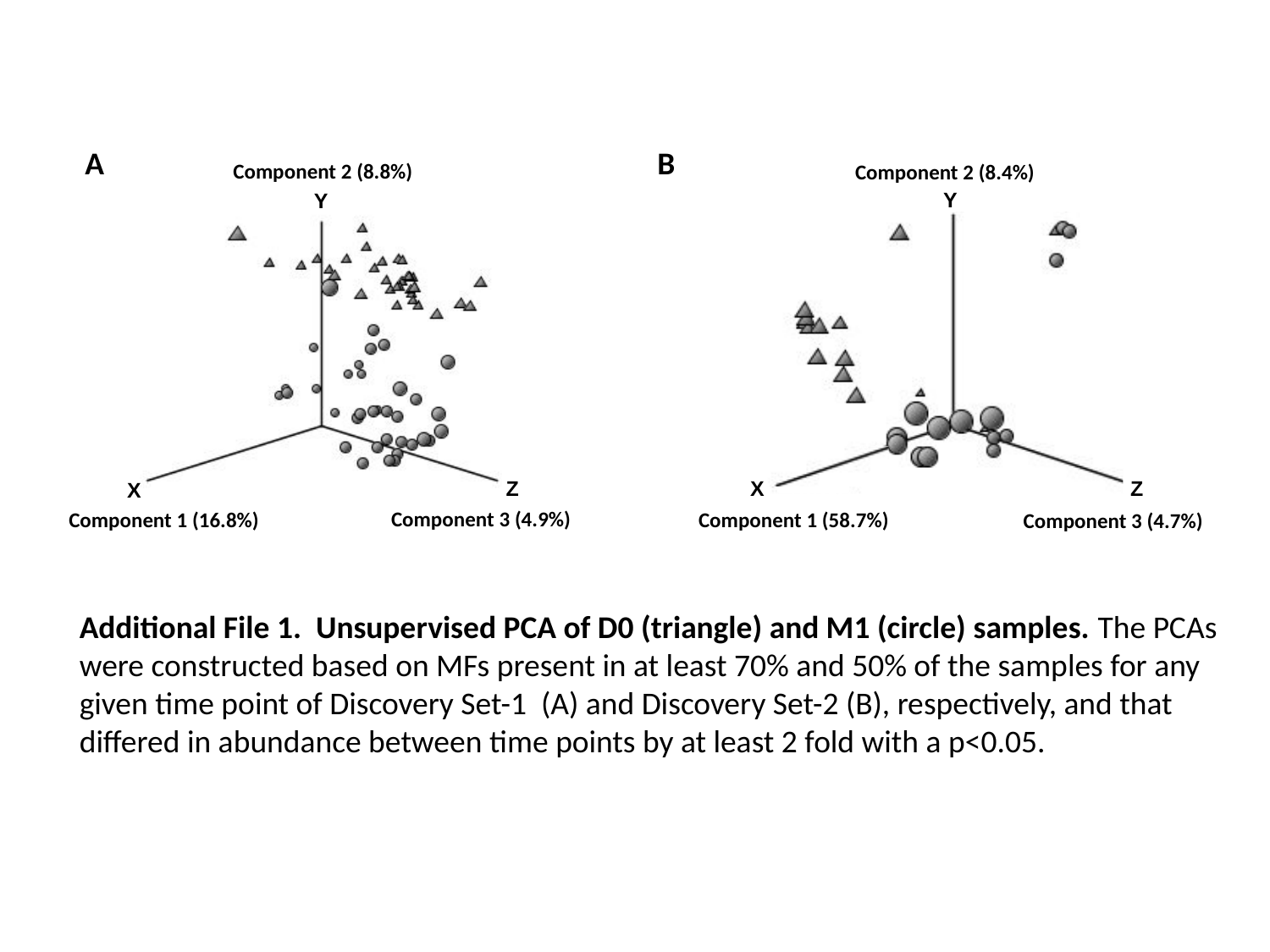

B
A
Component 2 (8.8%)
Y
Component 2 (8.4%)
Y
X
Z
Z
X
Component 3 (4.9%)
Component 1 (16.8%)
Component 1 (58.7%)
Component 3 (4.7%)
Additional File 1. Unsupervised PCA of D0 (triangle) and M1 (circle) samples. The PCAs were constructed based on MFs present in at least 70% and 50% of the samples for any given time point of Discovery Set-1 (A) and Discovery Set-2 (B), respectively, and that differed in abundance between time points by at least 2 fold with a p<0.05.
