# Supplementary material for: A metabolic biosignature of early response to anti-tuberculosis treatment
Source: BMC Infect Dis. 2014 Jan 31;14:53. doi: 10.1186/1471-2334-14-53 (PMC3918231; doi:10.1186/1471-2334-14-53)
Supplement: Additional file 3 — Per Patient Evaluation of MF Robustness. [file 1471-2334-14-53-S3.docx]

**Additional File 3. Per Patient Evaluation of MF Robustness**

| **Cluster^1^** | **MF^2^** | Percent Patient Change in Abundance^3^  (Decreased/Increased/Missing Data^4^) | | | | | |
| --- | --- | --- | --- | --- | --- | --- | --- |
|  |  | Qualification Set 1 | |  | Qualification Set 1 | | |
|  |  | **ΔD0-M1** | **ΔD0-M6** |  | **ΔD0-M1** | **ΔD0-M2** | **ΔD0-M6** |
| C1 | 287.1737 | 65/29/6 | 38/62**/**0 |  | 35/55/10 | 45/50/5 | 40/55/5 |
|  | 329.2206 | 65/35/0 | 58/38/6 |  | 35/65/0 | 40/60/0 | 40/60/0 |
|  | 681.3793 | 56/38/6 | 71/26/3 |  | 50/50/0 | 55/45/0 | 55/45/0 |
| C2 | 287.1736 | 62/32/6 | 56/44/0 |  | 45/55/0 | 65/35/0 | 50/50/0 |
|  | 311.2105 | 68/29/3 | 74/23/3 |  | 50/40/10 | 45/45/10 | 50/40/10 |
|  | 329.2574 | 79/18/3 | 79/18/3 |  | 55/45/0 | 55/45/0 | 60/35/5 |
|  | 610.3365 | 47/12/41 | 65/15/21 |  | 35/10/55 | 65/5/30 | 45/5/50 |
| C3 | 245.1636 | 59/41/0 | 59/41/0 |  | 45/55/0 | 80/20/0 | 65/35/0 |
|  | 285.1334 | 59/41/0 | 62/38/0 |  | 75/25/0 | 80/20/0 | 85/15/0 |
|  | 317.2208 | 68/29/3 | 43/47/0 |  | 35/60/5 | 55/40/5 | 50/50/0 |
| C4 | 137.0480 | 79/21/0 | 68/23/9 |  | 90/0/10 | 85/5/10 | 75/0/25 |
|  | 167.0591 | 74/26/0 | 82/18/0 |  | 75/25/0 | 80/20/0 | 85/15/0 |
|  | 170.1420 | 91/6/3 | 79/18/3 |  | 45/45/10 | 50/30/20 | 60/25/15 |
|  | 202.1326 | 91/9/0 | 94/6/0 |  | 95/5/0 | 95/0/5 | 95/5/0 |
|  | 231.1831 | 68/29/3 | 76/21/3 |  | 75/25/0 | 95/5/0 | 95/5/0 |
|  | 263.1124 | 91/9/0 | 88/12/0 |  | 85/10/5 | 95/5/0 | 75/25/0 |
|  | 359.2308 | 65/26/9 | 71/26/3 |  | 75/25/0 | 65/35/0 | 65/15/20 |
|  | 364.2243 | 65/21/15 | 59/18/24 |  | 50/50/0 | 45/55/0 | 70/30/0 |
|  | 496.2014 | 56/35/9 | 71/24/6 |  | 70/25/5 | 75/25/0 | 80/20/0 |
|  | 566.2683 | 71/21/9 | 65/26/9 |  | 65/25/10 | 80/10/10 | 80/10/10 |
|  | 688.2028 | 65/35/0 | 62/38/0 |  | 60/30/10 | 65/25/10 | 60/30/10 |
| C5 | 286.2374 | 68/32/0 | 85/15/0 |  | 90/10/0 | 80/15/5 | 65/15/20 |
|  | 421.2050^5^ | 85/12/3 | 59/9/32 |  | 90/10/0 | 95/5/0 | 95/5/0 |
|  | 583.2573 | 79/6/15 | 65/6/29 |  | 50/20/30 | 50/35/15 | 70/5/25 |
|  | 874.3500 | 88/9/3 | 91/6/3 |  | 90/10/0 | 90/10/0 | 95/5/0 |
| C6 | 159.1263 | 62/38/0 | 68/32/0 |  | 40/45/15 | 40/30/25 | 40/40/20 |
|  | 315.2421 | 71/29/0 | 68/32/0 |  | 35/65/0 | 55/45/0 | 85/15/0 |
| C7 | 200.1527 | 79/18/3 | 88/9/3 |  | 65/35/0 | 50/50/0 | 95/5/0 |
|  | 655.1932 | 88/9/3 | 85/12/3 |  | 65/35/0 | 75/25/0 | 90/10/0 |
| C8 | 246.0865 | 91/6/3 | 76/21/3 |  | 95/0/5 | 85/5/10 | 75/25/0 |
|  | 301.2267 | 71/29/0 | 71/29/0 |  | 40/60/0 | 40/60/0 | 40/60/0 |
| C9 | 313.2265 | 68/32/0 | 71/29/0 |  | 50/50/0 | 45/55/0 | 50/50/0 |
|  | 315.2050 | 71/29/0 | 74/26/0 |  | 40/60/0 | 20/80/0 | 40/60/0 |
| * | 174.0636 | 35/62/3 | 24/73/3 |  | 20/75/5 | 35/60/5 | 45/55/0 |
| * | 311.1239 | 53/44/3 | 78/29/3 |  | 75/25/0 | 75/25/0 | 85/15/0 |

^1^The designated cluster is defined in Figure 4

^2^ The MF is denoted by its monoisotopic mass.

^3^ Percent change in abundance was determined for D0 versus M1, M2, or M6

^4^The missing data indicates the percentage of patients for which the MF was not detected at one or more time points.

Highlight fields indicate MFs that consistently decreased in abundance from D0 in at least 60% of the patients for all time points of a sample group.
